# The Role of Nature in Coping with Psycho-Physiological Stress: A Literature Review on Restorativeness

**DOI:** 10.3390/bs4040394

**Published:** 2014-10-21

**Authors:** Rita Berto

**Affiliations:** Department of Education, Philosophy and Psychology, University of Verona, via San Francesco 33, 37129 Verona, Italy; E-Mails: rita.berto@hotmail.it; rita.berto@univr.it; Tel.: +39-339-640-2891; Fax: +39-045-802-8465

**Keywords:** stress, coping strategy, restorative environments, stress recovery, attention restoration, Perceived Restorativeness Scale, urban green

## Abstract

Physical settings can play a role in coping with stress; in particular experimental research has found strong evidence between exposure to natural environments and recovery from physiological stress and mental fatigue, giving support to both *Stress Recovery Theory* and *Attention Restoration Theory*. In fact, exposure to natural environments protects people against the impact of environmental stressors and offer physiological, emotional and attention restoration more so than urban environments. Natural places that allow the renewal of personal adaptive resources to meet the demands of everyday life are called *restorative environments.* Natural environments elicit greater calming responses than urban environments, and in relation to their vision there is a general reduction of physiological symptoms of stress. Exposure to natural scenes mediates the negative effects of stress reducing the negative mood state and above all enhancing positive emotions. Moreover, one can recover the decrease of cognitive performance associated with stress, especially reflected in attention tasks, through the salutary effect of viewing nature. Giving the many benefits of contact with nature, plans for urban environments should attend to *restorativeness*.

## 1. Introduction

This brief review attempts to draw greater attention to the role of the physical environment in stress recovery and psychological restoration processes. As people interact daily with physical settings, the physical environment can heighten stress or help people cope with it. “Stress” can be defined as the condition that results when person-environment transactions lead the individual to perceive a discrepancy (whether real or not) between the *demands* of a situation and the biological, psychological or social *resources* of the individual [1]. The negative effects of stress can be measured in various ways inside and out of the laboratory and these measures fall into three categories: those that rely on (1) neuro-physiological or bodily changes in the individual experiencing stress, (2) performance or behavioral changes and (3) self-report by individuals. The present paper updates and reviews the literature from each area, discussed separately.

In Psychology, most theoretical accounts of stress effects invoke some variation of the inverted U-hypothesis relating arousal to performance [2], with both high and low levels of arousal causing reduced performance. However, other theories account for effects of stress on cognition, specifically on attention allocation, with stress overloading attentional capacity for the deployment of attention itself and other resources [1]. Except for social support (operationalized primarily in terms of family, socio-cultural and economic conditions), Health Psychology has directed attention away from environmental factors that can be coping resources, whereas research and theories in Environmental Psychology point also to certain kind of environments that have the capacity to facilitate recovery of depleted resources. Environmental conditions are antecedent factors in stress-related mechanisms interceding between environment and health: they might operate as an environmental stressor, straining human adaptive capacities, or as a coping strategy, re-establishing some balance between environmental demands and personal resources. People rarely respond to stressful conditions passively, instead they use coping strategies. The physical environment can *damage* or *ameliorate* coping resources, thus heightening or reducing stress themselves.

## 2. The Mechanisms and Theoretical Approaches Underlying the Phenomenon

Experimental research has found evidence that restoration from stress and from mental fatigue relates to exposure to nature [3,4]. Natural environments protect people against the impact of environmental stressors and offer physiological, emotional and attention restoration more so than do urban environments. Natural places that allow a shift towards more positively-toned emotional states, positive changes in physiological activity levels, and in behavior and cognitive functioning are called *restorative environments* [5,6]. For pragmatic and theoretical reasons [7] nature scenes dominated by green vegetation have been the most frequently studied among restorative environments, with a relatively high success rate [8]. Exposure to nature is a coping strategy, which has positive effects on both arousal/activation level and cognitive overload. *Arousal* theories [9,10] imply that recuperation from excessive arousal should occur more rapidly in settings having low levels of arousal. Since natural settings tend to have lower levels of arousal properties, such as complexity, intensity and movement than urban environments [11], they should have comparatively restorative influences on stress. The alternative *overload* perspectives provide a different explanation of why recuperation following a stressor may be more rapid when external stimulation is comparatively low. High complexity and other increasing-stimulation properties typical of urban settings, place taxing processing demands [12] and elicit more sustained attention than nature settings; accordingly restoration from cognitive overload is hampered.

Research on restorative environments has developed within two complementary theoretical positions: the *Stress Recovery Theory* (SRT) [13] and the *Attention Restoration Theory* (ART) [6]; the former is a psycho-evolutionary theory, while the latter a psycho-functionalist. The *evolutionary* perspective contends that because humans evolved over a long period in natural environments, people are to some extent physiologically and perhaps psychologically adapted to natural, as opposed to urban settings [4]. To *functionalist,* humans have an unlearned predisposition to pay attention and respond positively to natural content (e.g., vegetation, water) and to configurations characteristic of settings that were favorable to survival during evolution [5,13,14,15]. Although in both theories natural environments are more restorative than urban or artificial environments, they differ in what drives individuals toward a restorative place: In SRT it is *physiological stress*, whereas in ART it is *mental fatigue*. These theories complement one another, in that the elevated physiological arousal and negative affect characteristic of stress (SRT) can occur in absence of mental fatigue. Conversely elevated arousal or negative affect do not always accompany attentional fatigue (ART); attentional fatigue can be considered a stress aftereffect and treated as a condition that increases vulnerability to stress [3,4,6,12,16].

Research related to these two theories agrees on two findings: (1) Environmental preference is affected by people’s need to get restoration [17,18,19,20,21,22]; (2) Environments perceived as natural tend to be more restorative than environments perceived to be urban or artificial e.g., [3,23,24,25,26,27,28]. In fact, research shows restoration related to environmental preference, but the direction of the effect remains unclear; however the positive linear correlation between the perception of place restorative qualities and environmental preference may suggest that the general preference for natural environment can be explained by individual conviction that “psycho-physiological” restoration occurs easier in natural environments. In practice people prefer natural environments because those places allow maintaining or enhancing psycho-physiological wellbeing.

*Mental fatigue* gives higher preference for the natural over the urban environment [22]. Nature is especially conducive to our *involuntary* attention engagement; on the contrary built content captures attention dramatically, requiring attention to be overcome [6,8,24]. In the ART this *attention-drawing quality* of natural settings is referred to as “soft fascination” [6]. When nature captures people’s attention, the executive system that regulates directed attention gets to rest, pessimistic thoughts are blocked, and negative emotions are replaced by positive ones [29]. In addition to fascination, nature is characterized also by other properties called *restorative factors*, which work together with fascination. Actually, the renewal of a depleted capacity also occurs with a physical and/or psychological “being-away” from demands on directed attention, a sense of “extent”, *i.e.*, being in a large enough world where “coherence” and “scope” are perceived in the environment, and “compatibility” between one’s inclinations and the environmental demands, for more details see [6,21,23].

The research does not claim that the restorative experience can occur only in natural environments, nor does it state that all urban environments lack restorative qualities [30,31]. For example, some natural environments would not likely be restorative because they are perceived as dangerous [32,33], and some urban environments, such historical environments [18], museums [34], or monasteries [35], can sustain restoration because they have to some extent restorative qualities, are easily approachable and so compatible with the little free time of the majority of the inhabitants of the city [36,37]. Accordingly the involuntary attentive process might be activated also by the vision of urban environments [31], but only if environmental information is *fascinating*, *i.e.*, doesn’t overload the attentive system [38], as nature does. ART claims that fascination is a restorative characteristic of an environment related to information processing, therefore suitable urban-artificial solutions can sometime fill the gap due to the lack of nature [30,31,39].

## 3. The Direct and Indirect Effects of Natural Environments

Central to the recovery from psycho-physiological stress are positive changes in emotional states. Exposure to natural environments produce positive mood chances, actually exposure to natural stimuli can mediate the negative effect of stress reducing the negative mood state and at the same time enhancing positive emotions. In particular, natural settings have restorative influences on three affective dimensions: positive affects, anger/aggression, and fear [4,40,41]. Moreover, people report more positive emotions (such as friendliness) and fewer negative emotions (such as sadness) when viewing urban scenes with trees than when viewing the same scenes with inanimate objects [42]. In contrast, exposure to environments lacking of natural elements can produce anxiety, anger, frustration and sadness [43,44]. The association between environment and emotions leads people to assess natural environment on the opportunity they offer to regulate mood, in practice preference for natural environments arises from the favourable effects on mood of such environments [45].

The positive emotional states elicited by viewing natural stimuli are part of the mechanism underlying the landmark Ulrich’s [46] finding that hospital patients had more favorable recovery (shorter post-operative hospital stays, lower scores post-surgical complications, fewer negative comments in nurses’ notes, fewer strong analgesic intake) if their windows overlooked trees rather than a brick building wall. Exposure to nature can reduce anxiety, improve pain control and patients’ satisfaction with the procedure [47,48]. One study found that the exposure to natural environments (pleasant stimuli) effectively distracted patients from stressful or painful conditions [47]. Another study found that heart-rates and self-reports of emotional state of patients in dental clinics improved with exposure to natural environments [49]; patients felt calmer on days when a mural depicting a natural scene was on the wall then on days when the wall was blank. To this end, Diette *et al.* [47] recommended the routine clinical use of nature sights and sounds of sights. The use of nature scenes was shown to be an effective tool for “distraction”, *i.e.*, patient’s attention is focused on a pleasant stimulus and away from a stressful or painful condition. Research in prisons has shown that prisoners whose cell windows offered views of farmland and trees had lower frequencies of stress symptoms such as digestive illness and headaches, and fewer sick calls than prisoners whose cell windows offered views of the prison courtyard [50,51].

The negative effects of psycho-physiological stress can also manifest with significant decreases of cognitive performance. However, people can recover cognitive efficiency simply taking advantage of the beneficial effect deriving from exposure to nature. For example, children playing in highly natural school playgrounds showed fewer attention and concentration problems, and improved cognitive and physical functioning than children playing in less natural school playgrounds, for a review [52]. At workplace, a view of natural elements was found to buffer the negative impact of job stress, intention to quit and it had a positive effect on general wellbeing and cognitive functioning [53,54]. The most significant understanding of nature’s salutary effect on cognition comes through studies of attention. Research has shown that natural settings might have restorative effects that include increased performance on task requiring attention and cognitive processing [5,23,55,56,57]. Cognitive restoration following visual exposure to the natural environment, as reflected in improved performance on attentional tasks, has been established in a variety of experimental studies involving either the use of videos [17] or actual field trips [3,58,59], or image slideshows of natural scenes [23,55]. Kaplan’s ART [6] gives a convincing explanation of what makes up the so-called “psychological restoration.” The theory originated when it was noticed that people preferred scenes depicting natural than urban environments, and exposure to natural environments had a profound restorative effect on the ability to focus, in practice people’s attention was easily and almost effortlessly held. The tenets of this theory state that a person can engage two types of attention: *involuntary* and *voluntary*, for more details see [60]. The former is a rather effortless form of attention, in contrast the latter, otherwise called *directed attention*, requires a good deal of focus and effort that leads inevitably to *mental fatigue.* The mental fatigue state increases the probability that an individual experiences the stress response due to the cognitive overload, and the concomitant reduction of the cognitive resources necessary to address daily requests. Mental/attentional fatigue manifests itself in negative emotions, irritability, impulsiveness, impatience, reduced tolerance for frustration, insensitivity to interpersonal cues, decrease altruistic behaviors, reduced performance, increased likelihood of taking risks [58,61,62,63,64], generally speaking in reduced competence and/or decreased effectiveness in functioning [58,63]. In practice, the inability to renew the attentional capacity aggravates the mental fatigue state and can also damage mood, work performance and interpersonal relationships.

Nature may not only have direct effects on stress recovery and mental fatigue restoration, but it may also have indirect effects by serving as a *buffer* against the health impacts of stressful events [65]. Many people seek out nature in time for stress. Unfortunately due to increasing urbanization, modern people’s homes have become more removed from green environments [65]. Restricted access to green spaces may increase people’s vulnerability to the impact of stressful life events and environmental stressors affecting physical and psychological wellbeing. Higher accessibility to park/forest-like area correlates with higher happiness, lower stress, anger, depression and tension, improved mood and concentration [17]. In particular, the amount of green space within a radius of 1–3 km relates to perceived general health [66].

Thus, urban green besides making our cities more appealing, gives relief from stressful life, and an opportunity to recover cognitive resources and restore the optimal level of physiological activation [5,13,44,67]. This can have positive effects on sense of *control*, *privacy*, encouraging *personal relationships* and *physical exercise*, and offering natural *fascinating distractions* that promotes positive emotions and mood. Loss of control and the lack of privacy can aggravate the stress condition and threaten individual’s capacity to cope with stressful situations [68]. Exposure to nature offers the opportunity to display control through a “temporary being-away” or “temporary escape” from reality. Estrangement from habits/routines means to go away from the source of stress. Regarding social support. Outdoor spaces and gardens can promote social relationships and enhance the sense of community. Mental health services engage nature-related programs (horticulture, gardening) to provide opportunities that enhance multiple aspects of health and wellbeing, increase constructive interpersonal relationships that enhance social inclusion, and support the destigmatization of mental illnesses [69,70]. Participants benefit from the increase of positive emotions, expand healthy relationships with peers and staff, improve physical activity, have greater involvement in familiarity within the community and exhibit skills that enable acceptance in the community and the perception of being part of the community [71]. Active participation in nature has additionally been found to reduce mental distress, enhance self-confidence and improve physical health of the participants [72].

The recognition of nature’s health benefits has brought to broader discussion on public health and also inspired practical applications. In particular psychologists have begun to study whether technology can salvage some of nature’s healthful properties [73]. Although virtual nature may not replace actual nature, people who are not able to go outside can benefit from exposure to virtual nature [74]. When real nature is not at hand, surrogate (artificial plants, potted plants) or simulation (pictures of nature, films, slides) of nature are accepted at work, in hospitals or institutions providing restorative effects such as improved affect and decreased physiological stress [17,75]. In particular, the immersion in a virtual computer-generated nature setting has been found to be a valid therapeutic aid in treatment of anxiety disorders and an effective tool in stress management and relaxation [76,77]. Virtual settings are enriched with a variety of positive visual and auditory stimulation that affect self-efficacy and mood; the virtual reality (VR) scenario-experience is vivid and real and induces a high sense of “presence” that affects relaxation and the emotional response.

## 4. How to Measure the Effects of Natural Environments

### 4.1. Physiological Effects

Though their concern was not to compare the effects of natural *vs.* urban settings, back in 1963 Wadeson *et al.* [78] found evidence that exposure to natural environments had a direct influence on urine and blood levels of cortisol, a stress-related hormone. More recently, literature has shown that independently from the type of exposure: plants, poster, slides, video, VR settings or views of natural environments/stimuli, people experience a general reduction of symptoms related to psycho-physiological stress. Natural environments elicit greater calming physiological and psychological responses than urban environments. The SRT [13] proposes exactly that perceiving particular qualities and contents in a place can support recovery from physiological stress. Using a paradigm in which stressed individuals were exposed to simulations of either natural or urban environments, Ulrich encompassed the range of restorative effects of the natural environments on human beings [4,13,46,79]. Research showed different rates of recovery from stress depending upon the type of environmental exposure. Physiological measures of stress (e.g., electromyography, skin conductance response, pulse transit time, cardiac response, partial thromboplastin time) indicated that recovery was quicker and more complete in the natural environment exposure conditions, even when recovery was measured over a 10-minute period only [79]. In the initial minutes of recovery the parasympathetic component response was recorded to the natural environments, whereas there was no evidence of the parasympathetic involvement in response to the urban settings. The parasympathetic system, often called “relax and renew,” is the branch of the Autonomic Nervous System (ANS) responsible for recuperating and returning to a balanced state (homeostasis) after experiencing a stressful situation; it reacts to return the body to a state of equilibrium by slowing down heart rate, dilating blood vessels, activating digestion, and storing energy. In contrast, the sympathetic system, the other branch of the ANS, activates in response to stressors; it is also known as the “fight or flight” response [80], because its activation is central in the taxing mobilization involved in responding to unexpected stressful events.

The relaxing effect of nature is supported by electroencephalogram (EEG) data as well. EEG measures are sensitive to conditions such as fatigue and sleep deprivation. If so, perhaps neuro-physiological measures, such as the EEG or functional magnetic resonance imaging (fMRI), might be used to differentiate stress states of the organism from normal or restored states. Unstressed subjects who viewed slides of natural landscapes and urban scenes [79], or single natural elements such as plants with flowers and pots without plants [81], or who were seated in an outdoor setting watching greenery or a concrete block fence [82] had greater brain electrical activity in the alpha frequency range. High alpha amplitude is associated with lower level of physiological arousal as well as feeling of wakeful relaxation [79]. Generally, feeling of anxiety are related to high arousal and accordingly to low alpha amplitude. All these results suggest that subjects are less aroused physiologically and more relaxed but wakeful, during exposure to natural stimuli. EEG studies identify tranquility as an outcome of viewing natural settings [83]. Recently, Korean researchers used the fMRI to investigate brain activation patterns in participants viewing nature *vs.* urban scenes [84]. The urban scenes showed enhanced activity in the amygdala, which is linked to impulsivity, anxiety and increased stress. By contrast, the natural scenes promoted activity in the anterior cingulate and the insula – where increased activity is associated with heightened empathy and altruistic behavior.

### 4.2. Behavioral Effects

A logical extension of attention restoration theory is that people deprived of nature will display behaviors caused by weary minds: inhibition is essential to delay and reflection, lacking this capability an individual behaves in a less adaptive and appropriate fashion [6]. Moreover, without the patience and endurance necessary to carry out difficult or unpleasant tasks, performance becomes more oriented to the short term. In fact, directed attention fatigue not only leads to the inability to focus, but it has also several unfortunate consequences, including performance errors, inability to plan, social incivility and irritability [6]. Taylor, Kuo and Sullivan [85] found also a relationship between exposure to nature and self-control; in studying a group of girls living in the same housing complex, the researchers found that those with greener views scored higher than those deprived of nature on several tasks related to discipline, higher concentration, inhibited impulsivity and ability to delay gratification. Regarding social behavior, which also depends upon inhibition, it becomes less appropriate and there is also a greater inclination to be impulsive, to take unnecessary risks, and to act in an impatient and hasty manner. Kuo and Sullivan [86] reported significantly lower levels of aggression and violence in residents with apartments near nature than in those who looked onto barren lands; the researchers suggested that if fatigued attention is related to irritability, and irritability leads to impulsivity and aggression, then perhaps people deprived of nature’s restorative qualities would be overly aggressive. In general, exposure to nature enhances sense of attachment, social life, mental and physical health, quality of life and the occurrence of activities and events that enhance wellbeing. In particular, green vegetation in neighborhood common spaces correlates with stronger ties, higher sense of safety and adjustment [87], less aggressive behavior, and fewer property and violent crimes reported to the police than areas without greenery [86].

Views of nature affect driving as well. Comparing the physiological responses of subjects who watched a video driving through nature with those who watched a drive through more built-up environments, Parsons *et al.* [88] found that the nature-group displayed lower levels of stress and recovered more quickly from the stress they experienced. Views of dense vegetation (vs. sparse and mixed) enhance in fact drivers’ ability to tolerate frustration [89].

### 4.3. Self-Report Measures

Quantifiable measures of restoration are the key to understand how restorative mechanisms work, and research on the buffering effects of nature among stressed or mentally fatigued individuals has mostly relied on physiological and cognitive measures as outcome variables. However, together with physiological, behavioral and performance measurements there are also self-report measures aimed to assess the restorative value of real places/pictures, *i.e.*, the degree of *perceived* *restorativeness* of a setting. To this aim, the majority of the studies have used the *Perceived Restorativeness Scale* (PRS). The scale based on the ART, which appeared in 1997 [25,26], aimed to measure the presence of the four theoretical restorative factors (being-away, fascination, extent, compatibility) in the environment. From then on, the PRS has been widely used not only to compare the restorative value of natural and urban settings, but also to measure perceived restorativeness of outdoor activities [90,91], vacation destination [92], zoo and small public parks [93,94,95], and it has appeared with different names, e.g., Restorative Outcome Scale [96], Revised Perceived Restorativeness Scale [97], Perceived Restorative Characteristics Questionnaire [94], PRS-short version [23], but questions about the scale validity/reliability remain.

Since the PRS appearance researchers have differed on the number of items making up the scale and on its factorial structure [97,98]. However, research has confirmed, through the PRS, both the positive correlation between environmental preference and perceived restorativeness, and the lack of correlation between familiarity on perceived restorativeness [21,99]. Furthermore, research using the PRS found that the higher restorative value of natural *versus* urban or artificial settings did not differ with gender or age [100]. Primary school children can discriminate the restorative value of environments varying in their degree of naturalness [101], and assess natural environment more restorative than school environments and playground [102].

Regarding the PRS factorial structure, Pasini *et al.* [103] have shed light on the psychometric characteristics of the scale. After a detailed understanding of the meaning of PRS individual items with the method of cognitive interviews, which is the proper starting point for the development of a self-rating scale, Pasini *et al.* [103] ended up in an 11-item. Using Confirmatory Factor Analysis to compare five models based on previously published research and underlying theory, the researchers found that a four-factor model that mirrored the four factors of ART, had the best fit to the data. The resulting 11-item PRS was also invariant across nationality and gender.

The PRS-11 is less concerned with people’s environmental preference. Instead, it addresses the perceived “cognitive” supportiveness of the environment in relation to individual’s psychological wellbeing. It assesses aspects of purposive behaviors to avoid mental fatigue, and the cognitive fit between person and environment is indicative of a “no-mental fatigue” state in relation to the environment, and not only of how an environment is restorative. For this reason the PRS-11 can help researchers and community planners who can both rely on a valid, reliable, brief and easy to comprehend instrument. In fact, for people interested in people’s wellbeing, it is the subjective fit which is essential, *i.e.*, the perceived supportiveness of the environment in connection with the personal goal to recover from mental fatigue.

The PRS-11 assumes that people’s subjective appraisal of their environments provides a reasonable, straightforward index of the quality of their psychological restoration experiences in those settings. However the complex psycho-physiological pathways of stress make measurement via one single measure insufficient. Moreover the issue of which type of measure (behavioral, self-report or neuro-physiological) is the better or more appropriate measure of stress effects is far from settled. Stress impacts physical and mental health and a number of inter-personal differences have been found to impact on the ways we experience and interact with green space, they are gender [104], age [105], culture/ethnicity [106], interests/expertise [107] and it is a matter of fact that different “extraneous” variables are associated with restorative experiences in favorite settings [96]. Nevertheless, the relationship between perceived restorativeness and stress measures has not been firmly established. To this end, considering the different kinds of measures (self-report, physiological, behavioral, task performance) a multi-method multi trait study (MMMTS) would allow assessing the adequacy, namely if the PRS-11 fits with other measures of stress or recovery. However, the conceptual formulation of the trait “perceived cognitive supportiveness/perceived restorativeness” implicitly includes the proposition that this trait can be meaningfully differentiated by other traits.

## 5. Conclusions

Given the many benefits from contact with nature, plans for urban settings should consider the human need for restoration. For this, research must offer practical guidelines for the accessibility and quality of urban green areas. A well-designed urban landscape can contribute to creating a less stressful day [108] and to providing an opportunity for physical, cognitive and emotional restoration [36]. Thus, research can help integrate natural elements and structural features into built environments [109] in order to plan urban environments that are “cognitive sustainable” and restorative from mental fatigue and the stresses of urban life [30,110].

Empirical evidence on the stress reduction from exposure to natural settings agrees with both SRT and ART. However, the findings are unclear about whether active or passive involvement with nature is preferable for restorative benefits, and whether restorative outcomes (both physiological and cognitive) vary with the length of exposure to natural stimuli. According to adaptation level theory [111,112] people adapt (or get accustomed) to their environments. If that applies to exposure to natural environments, then people surrounded by nature might require a higher dose (a sort of threshold) to recover from stress and mental fatigue than would people surrounded by buildings. On the other hand, their long-term exposure may inoculate them from stress. Research could also consider effects related to different kinds and form of nature, as well as individual and cultural differences in the perception of restorativeness and the restoration process.

Another important issue concerns the relation between biophilic design and psychological wellbeing, *i.e.*, whether the presence of natural features (such as curvilinear forms, gradations of colors, blending of textures) and elements (water, plants) in buildings can have real benefits on human emotional wellbeing, stress reduction, cognitive efficiency, learning and healing processes.

Research on restorative environments has included both field and laboratory studies using primarily the transversal design, or to a lesser extent the pre-post design [113]. These studies miss a longer-term question. Successful coping strategies can mask the negative effects of a stressor. Studies have not determined whether long-term exposure to nature helps one adapt to or recover from stress/mental fatigue. To answer that question, we need longitudinal studies.
